# Control of Neuronal Migration and Aggregation by Reelin Signaling in the Developing Cerebral Cortex

**DOI:** 10.3389/fcell.2017.00040

**Published:** 2017-04-26

**Authors:** Yuki Hirota, Kazunori Nakajima

**Affiliations:** Department of Anatomy, Keio University School of MedicineTokyo, Japan

**Keywords:** neocortical development, neuronal migration, Reelin signaling

## Abstract

The mammalian cerebral neocortex has a well-organized laminar structure, achieved by the highly coordinated control of neuronal migration. During cortical development, excitatory neurons born near the lateral ventricle migrate radially to reach their final positions to form the cortical plate. During this process, dynamic changes are observed in the morphologies and migration modes, including multipolar migration, locomotion, and terminal translocation, of the newborn neurons. Disruption of these migration processes can result in neuronal disorders such as lissencephaly and periventricular heterotopia. The extracellular protein, Reelin, mainly secreted by the Cajal-Retzius neurons in the marginal zone during development, plays a crucial role in the neuronal migration and neocortical lamination. Reelin signaling, which exerts essential roles in the formation of the layered neocortex, is triggered by the binding of Reelin to its receptors, ApoER2 and VLDLR, followed by phosphorylation of the Dab1 adaptor protein. Accumulating evidence suggests that Reelin signaling controls multiple steps of neuronal migration, including the transition from multipolar to bipolar neurons, terminal translocation, and termination of migration beneath the marginal zone. In addition, it has been shown that ectopically expressed Reelin can cause neuronal aggregation via an N-cadherin-mediated manner. This review attempts to summarize our knowledge of the roles played by Reelin in neuronal migration and the underlying mechanisms.

## Introduction

The mammalian neocortex has a highly organized 6-layered laminar structure, which forms the basis of complex brain functions, including learning, memory, and cognition. During mouse neocortical development, excitatory neurons are generated near the ventricular wall from three types of progenitor cells: radial glia (RG) (Miyata et al., 2001; Noctor et al., 2001) and short neural progenitors (also called apical inter progenitor cells) (Gal et al., 2006) in the ventricular zone (VZ), and the intermediate progenitor cells, also called basal progenitors (BPs), in the subventricular zone (SVZ) (Noctor et al., 2004). RG have a long ascending (basal) fiber and a short apical process, and undergo interkinetic nuclear migration followed by mitosis at the apical surface (Miyata et al., 2001; Noctor et al., 2001). During neurogenic divisions, the RG exhibit asymmetric self-renewing division to generate neurons or BPs. Short neural progenitors possess an apical process and a basal process of variable length that is retracted during mitotic division, and also undergo interkinetic nuclear migration (Gal et al., 2006). Short neural progenitors undergo symmetric self-consuming division generating two neurons. BPs undergo terminal symmetric division in the SVZ to produce two neurons. Proliferation in the SVZ has expanded evolutionarily in primates. The primate SVZ is thick, consisting of an inner SVZ and outer SVZ (Smart et al., 2002). The human outer SVZ contains outer SVZ radial glia-like (oRG) cells, which possess an ascending basal process and undergo self-renewing asymmetric division (Fietz et al., 2010; Hansen et al., 2010). Subsequently, oRG-like BPs with the morphological features of radial glia were also found in other mammalian species, including rodents. These cells also exist in the inner SVZ of ferrets and marmosets, and are thus called basal RG (bRG) (Englund et al., 2005).

**Figure 1:**
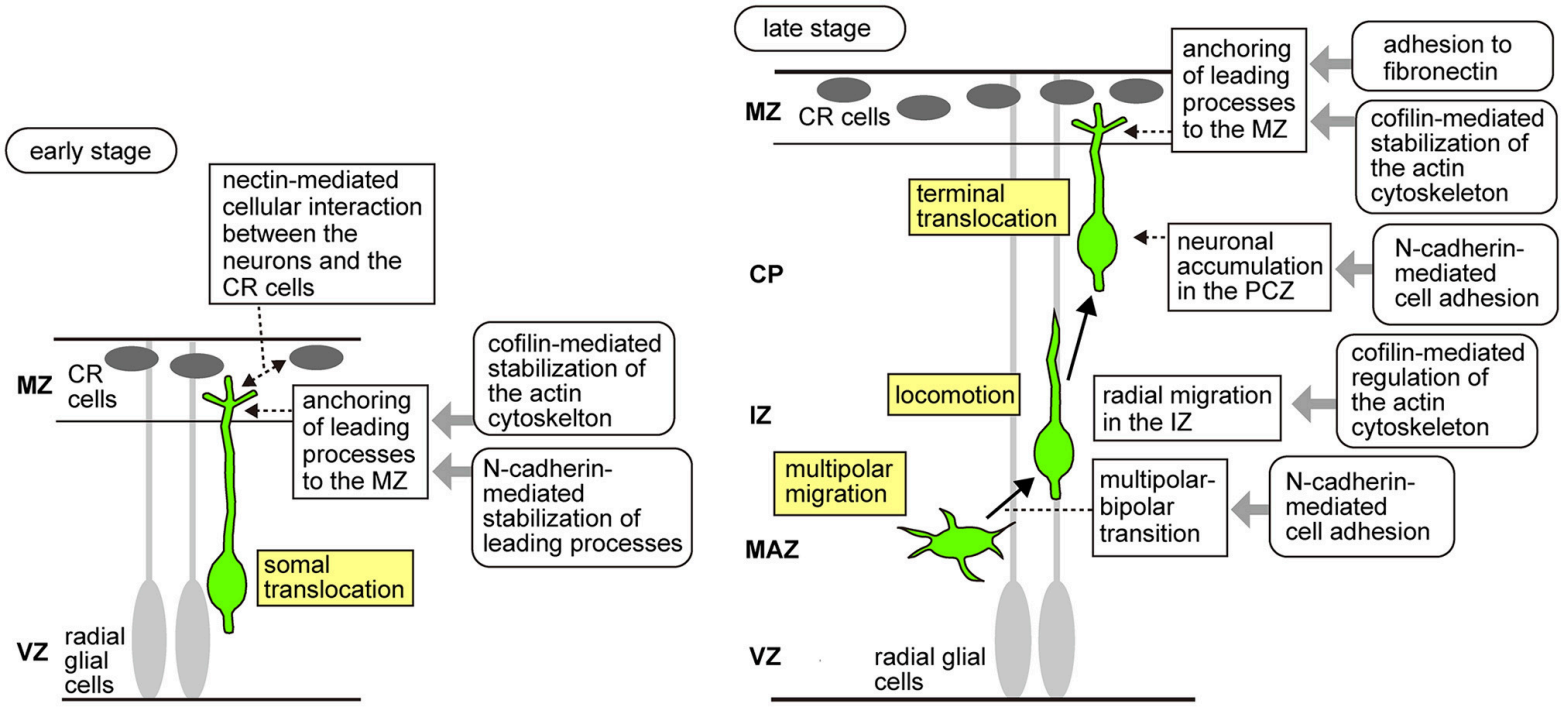
**Schema of the migratory modes of the excitatory neurons during neocortical development and molecular mechanisms downstream of Reelin signaling that control neuronal migration**. In the early developmental stages **(Left)**, neurons generated from the radial glial cells undergo somal translocation, in which they extend processes to the MZ and shorten their leading processes to move their cell bodies to their final positions just beneath the MZ. During somal translocation, nectin-mediated activation of N-cadherin promotes cellular interaction between the neurons and the CR cells. In addition, n-cofilin expressed in the migrating neurons promotes anchoring of the leading processes to the MZ. N-cadherin also stabilizes the leading processes of the migrating neurons elongating into the MZ. In the later stages **(Right)**, the neurons sequentially change their migratory modes. First, in the MAZ, they exhibit multipolar migration, in which they extend and retract multiple processes dynamically while their somata wander. Multipolar cells then transform into a bipolar morphology. Reelin signaling controls the multipolar-to-bipolar transition via the Rap1/N-cadherin pathway. N-cofilin-mediated regulation of the actin cytoskeleton is involved in the radial migration of the neurons in the IZ. Bipolar neurons show locomotion, in which they directionally migrate in the IZ and CP using radial glial fibers as the scaffold. Finally, when they arrive at the outermost region of the CP, they switch to the terminal translocation mode, in which the somata move rapidly in a radial glia-independent manner to just beneath the MZ to complete migration. N-cadherin promotes neuronal accumulation in the PCZ. Reelin signaling activates integrin α5β1 in the migrating neurons, causing them to adhere to the fibronectin localized in the MZ. LIMK1/n-cofilin and integrin α5β1/fibronectin promote anchoring of the leading processes to the MZ.

Newborn neurons exhibit multiple migration modes (Figure 1). At the beginning of cortical development, the earliest-born neurons form the preplate, a layer of differentiated neurons above the ventricular zone. Neurons subsequently generated in the VZ invade and split the preplate, which results in the formation of two distinct layers: the superficial marginal zone (MZ) and the deep subplate. Then, newborn neurons migrate radially from the VZ, pass through the subplate, and stop beneath the MZ to form the cortical plate (CP). During the early stages of neocortical development, radially migrating neurons destined to become the future deep layer neurons extend leading processes to the MZ, and then shorten their leading processes to move their cell bodies to their final positions. This migration mode is called “somal translocation,” in which neurons migrate in a radial glia–independent manner (Nadarajah et al., 2001). During the later stages, the neocortex becomes thick and the neurons begin to migrate in several sequential migration modes. Newborn neurons first remain in the VZ as pin-like cells (Tabata and Nakajima, 2003; Noctor et al., 2004; Tabata et al., 2009, 2012, 2013), and then move out of the VZ toward the brain surface to transform into multipolar cells. The multipolar cells remain just above the VZ, a region called the multipolar cell accumulation zone (MAZ), for about 24 h. In the MAZ, neurons show a unique behavior designated as “multipolar migration,” in which they extend and retract multiple processes dynamically while their somata wander (Tabata and Nakajima, 2003; Noctor et al., 2004; Tabata et al., 2009, 2012, 2013). Then, the multipolar cells transform into a bipolar morphology and start directed migration in the intermediate zone (IZ) and CP using the fibers of the RG as the scaffold (Rakic, 1972; Nadarajah et al., 2001). This RG-dependent migration is called “locomotion” (Nadarajah et al., 2001). Locomoting neurons directionally migrate toward the brain surface and when they arrive beneath the primitive cortical zone (PCZ), the outermost cell-dense region of the CP, they transiently pause, switching their migration mode to the “terminal translocation” mode (Sekine et al., 2011), which is morphologically similar to the somal translocation mode. Neurons use terminal translocation as the final migration mode, in which the somata move rapidly in a RG–independent manner to reach just beneath the MZ (Nadarajah et al., 2001) and complete their migration. During these processes, the later-born neurons pass through the early-born neurons at the top of the CP, resulting in a birth-date-dependent “inside-out” pattern, in which the later-born neurons are positioned in the superficial layers, while the early-born neurons reside in the deeper layers of the CP. This “inside-out” arrangement is specifically observed in the mammalian neocortex, suggesting its important roles in the expansion of the neocortex and evolution of the highly complex structure of mammalian brains.

An evolutionally conserved extracellular glycoprotein known as Reelin is now known to be involved in various cellular events in the development and functioning of the mature central nervous system. The most well-known function of Reelin signaling is control of neuronal migration and layer formation during neocortical development. In this review, we focus on the recently described roles of Reelin signaling in neuronal migration and aggregation in the developing neocortex.

## Core components of reelin signal transduction

Reelin was first identified as the responsible gene for the *reeler* mouse, a spontaneous recessive-mutant mouse which shows severe neurological phenotypes, including ataxia and malformations of the cortical layer structure (Bar et al., 1995; D'Arcangelo et al., 1995; Hirotsune et al., 1995). Birth-date labeling analysis revealed that the cortical layers are roughly inverted in these mice (Caviness and Sidman, 1973; Mikoshiba et al., 1980; D'Arcangelo et al., 1995; Ogawa et al., 1995; Boyle et al., 2011), suggesting that Reelin regulates the proper inside-out formation of the mammalian neocortex. Reelin binds to the cell surface receptors apolipoprotein E receptor 2 (ApoER2) and very-low-density lipoprotein receptor (VLDLR), which belong to the low-density lipoprotein receptor family (D'Arcangelo et al., 1999; Hiesberger et al., 1999). Mice with double-knockout of *Apoer2* and *Vldlr* exhibited a roughly inverted laminar cortex, similar to the *reeler* cortex, whereas mice with knockout of either *Apoer2* or *Vldlr* alone showed weak phenotypes (Trommsdorff et al., 1999), suggesting that these genes have redundant functions. Reelin binding to its receptors results in phosphorylation of the intracellular adaptor protein, disabled 1 (Dab1), which binds to the cytoplasmic domain of the same receptors, by the Src-family kinases Fyn and Src (Howell et al., 1999). Mutant mice deficient in *Dab1* showed *reeler*-like phenotypes (Sweet et al., 1996; Sheldon et al., 1997; Ware et al., 1997; Yoneshima et al., 1997; Kojima et al., 2000). Furthermore, double-knockout mice for *Fyn* and *Src* and mutant mice with mutation of all the potential tyrosine phosphorylation sites of Dab1 also exhibit a phenotype similar to the *reeler* mice (Howell et al., 2000; Kuo et al., 2005), lending support to the idea that phosphorylation of Dab1 is required for Reelin signaling. Phosphorylated Dab1 recruits various molecules, including Crk/CrkL (Ballif et al., 2004; Chen et al., 2004; Huang et al., 2004), SOCS3 (Feng et al., 2007), Nckβ (Pramatarova et al., 2003), PI3K (Bock et al., 2003), and Lis1 (Assadi et al., 2003), and transmits the signal to downstream pathways. Dab1 has also been reported to shuttle between the nucleus and cytoplasm, suggesting its possible functions in the nucleus (Honda and Nakajima, 2006, 2016). Thus, a large number of molecules of the Reelin signaling pathway have been identified so far. In the following sections, we shall highlight the biological roles of Reelin signaling during neuronal migration.

## Roles of reelin signaling in preplate splitting and somal translocation

Preplate splitting is the first developmental event during cortical lamination that requires Reelin. In *reeler* mice, the CP neurons fail to split the preplate, resulting in a superficial “superplate,” composed of cells normally found in the MZ and subplate (Caviness and Sidman, 1973; Caviness, 1982), suggesting that somal translocation is impaired in the *reeler* mice. Subsequently arriving neurons accumulate beneath the superplate, resulting in an “outside-in” pattern of neuronal alignment, in which the later-born neurons are positioned in the superficial layers, while the earlier-born neurons reside in the deep layers of the CP (Caviness, 1982). Recent reports have shown that Reelin signaling controls somal translocation through distinct mechanisms. Chai et al. showed that Reelin signaling induced stabilization of the actin cytoskeleton during somal translocation. Reelin signaling, through LIMK1 activation, induces phosphorylation of n-cofilin, an actin-depolymerizing protein expressed in the leading processes of the neurons reaching the MZ, which promotes anchoring of these processes to the MZ (Chai et al., 2009). Another mechanism by which Reelin controls somal translocation is that which is mediated by neuronal cadherin, N-cadherin. Using *Dab1* flox mice, Franco et al. showed that Dab1 functions to stabilize the leading processes of the migrating neurons elongated into the MZ in a Rap1/N-cadherin-dependent manner during somal translocation (Franco et al., 2011). Cellular interactions mediated by Reelin signaling during somal translocation were also shown to be important. Gil-Sanz et al. focused on the roles of the immunoglobulin-like adhesion molecule, nectin. They showed that interaction between nectin1, expressed by the Cajal–Retzius (CR) cells, and nectin3, expressed by the migrating neurons, is critical for somal translocation. They also showed that Reelin signaling through the Crk/C3G/Rap1 pathway promotes N-cadherin functions via nectin3 and afadin, an adaptor protein which binds to the cytoplasmic domains of nectin, thus promoting heterotypic cell-cell interactions between the neurons and the CR cells (Gil-Sanz et al., 2013). Taken together, these results suggest that Reelin controls somal translocation via regulation of the actin cytoskeleton and cellular adhesion.

## Roles of reelin signaling in the radial migration of neurons in the IZ

In the developing cortex, Reelin is mainly secreted by the CR cells in the MZ (D'Arcangelo et al., 1995; Ogawa et al., 1995), however, a small amount of Reelin can also be detected in the lower part of the IZ (Yoshida et al., 2006; Uchida et al., 2009; Hirota et al., 2015). In addition, proteolytic processing of Reelin by neurons was shown to generate a small fragment of Reelin, which diffuses into the deeper part of the CP (Jossin et al., 2007). As for the Reelin receptors, ApoER2 is strongly expressed in the MAZ/lower IZ (Uchida et al., 2009; Hirota et al., 2015). Furthermore, using an alkaline phosphatase fusion protein of the receptor-binding region of Reelin, it was shown that functional Reelin receptors are mainly localized in the IZ/SVZ (Uchida et al., 2009). These findings suggest that Reelin binds to receptors expressed by neurons migrating in the deeper part of the cortex to exert its functions in their early migratory stages. Consistent with this notion, migration defect in the IZ is observed in the *Apoer2* KO and *reeler* mice (Hack et al., 2007; Britto et al., 2011; Hirota et al., 2016), while this radial glia-guided locomotion was not found to be altered when *Dab1* was genetically eliminated using conditional KO mice (Franco et al., 2011), suggesting that Reelin signaling is required for the migration step before locomotion. Lending further support to this idea, exogenous Reelin was shown to influence the migratory behavior of neurons in the VZ/SVZ in cultured brain slices (Britto et al., 2014). Reelin signaling seems to control neuronal migration in the IZ via N-cadherin-mediated cell adhesion. N-cadherin is required for proper polarization and directed radial migration of the neurons in the IZ (Gartner et al., 2012). Several lines of evidence suggest that cell surface expression of N-cadherin is regulated by endosomal trafficking mediated by Rap1, Rab, and Arf6 small GTPases during radial migration (Kawauchi et al., 2010; Jossin and Cooper, 2011; Hara et al., 2016). Expression of a dominant-negative form of the Reelin receptor VLDLR resulted in impaired multipolar-to-bipolar transition, possibly via the Rap1/N-cadherin pathway (Jossin and Cooper, 2011). Regulation of the actin cytoskeleton by Reelin also seems to be involved in the control of neuronal migration in the IZ. As mentioned above, Reelin signaling activates LIMK1, which results in enhanced n-cofilin activation (Chai et al., 2009). A recent study showed that migration defect in the IZ of *reeler* mice was partially rescued by overexpression of LIMK1 or a phosphomimic mutant of cofilin (Chai et al., 2016). These findings suggest that Reelin signaling influences neuronal migration in the IZ. Further studies are required to uncover how the low amount of Reelin protein expressed in the lower part of the IZ regulates this process.

## Roles of reelin signaling in terminal translocation

As mentioned above, Reelin is highly expressed in the MZ of developing cortex, and expression of Reelin receptors at the top of the CP and MZ has been repeatedly reported (Trommsdorff et al., 1999; Uchida et al., 2009; Kubo et al., 2010; Hirota et al., 2015). Since radially migrating neurons complete their migration beneath the MZ, existence of Reelin and its receptors in the MZ suggest a role of Reelin in the late steps of migration. Recent reports have shown important roles of Reelin signaling in terminal translocation. Sekine et al. showed that Reelin-dependent activation of neuronal adhesion to the extracellular matrix is crucial for the eventual birth-date-dependent layering of the neocortex (Sekine et al., 2012). Upon stimulation by Reelin binding to its receptors, Crk adaptor proteins bind to phosphorylated Dab1 and activate GTP exchange factor C3G, followed by activation of Rap1 GTPase. This pathway activates integrin α5β1, thus promoting the anchoring of the leading processes to the fibronectin localized in the MZ to promote terminal translocation (Sekine et al., 2012). Consistent with the morphological similarity between somal translocation and terminal translocation, some common mechanisms may be involved in the regulation of these processes. Reelin signaling has been shown to induce LIMK1/n-cofilin-mediated stabilization of the actin cytoskeleton and Rap1/N-cadherin-mediated stabilization of the leading processes during both terminal translocation and somal translocation (Chai et al., 2009; Franco et al., 2011). Together, these results suggest that Reelin signaling controls terminal translocation via regulating the actin cytoskeleton and cellular adhesion of the leading processes, and that both shared and distinct mechanisms are involved in the regulation of somal and terminal translocation during neuronal migration. It will be interesting to examine whether and how cellular interactions between migrating neurons and/or between neurons and cells in the MZ are involved in the regulation of terminal translocation by Reelin signaling.

## Ectopic expression of reelin induces neuronal aggregation *in vivo*

Recent findings described above indicate that Reelin signaling controls neuronal adhesion by regulating the adhesion of leading processes during translocation. Does Reelin signaling also control other aspects of migration and layer formation via controlling adhesion? Although locomoting neurons migrate individually through the IZ and CP, upon reaching the top of the CP, they seem to accumulate at a high cell density (Ajioka and Nakajima, 2005), suggesting the possibility that accumulation of the neurons in the PCZ may be required for proper layer formation through, for example, cell sorting among the neurons. Although the molecular and cellular mechanisms underlying the accumulation of neurons in the PCZ remain unknown, several lines of evidence suggest involvement of the Reelin signal. When Reelin was overexpressed in the IZ by electroporation into migrating neurons, the ectopically expressed Reelin caused the leading processes of the migrating neurons to assemble in the Reelin-rich region, resulting in ectopic neuronal aggregation *in vivo* (Kubo et al., 2010, Figure 2). Transplantation of Reelin-expressing cells into the developing cortex also yielded the same results. The center of the aggregate became cell-body-sparse and dendrite-rich, resembling the structure of the MZ, while the cell bodies were densely packed in the peripheral region of the aggregate, resembling the structure of the PCZ. In addition, sequential labeling of the early and late-born neurons revealed that during the formation of an aggregate, the late-born neurons pass by the early-born neurons to arrive at the cell-body-sparse center of the aggregate (Kubo et al., 2010). It was shown that ectopically expressed Reelin caused neuronal aggregation via binding to the ApoER2 expressed in the migrating neurons, as inhibition of ApoER2 disrupted the aggregate formation (Kubo et al., 2010; Hirota et al., 2016). These results indicate that ectopic Reelin is responsible for the birth-date-dependent “inside-out” pattern of neuronal alignment even in the ectopic cellular aggregates, similar to that which would occur beneath the normal MZ. Thus, this ectopic expression model serves as a useful tool to investigate the roles of Reelin signaling in the cell accumulation or aggregation at the final step of neuronal migration.

**Figure 2:**
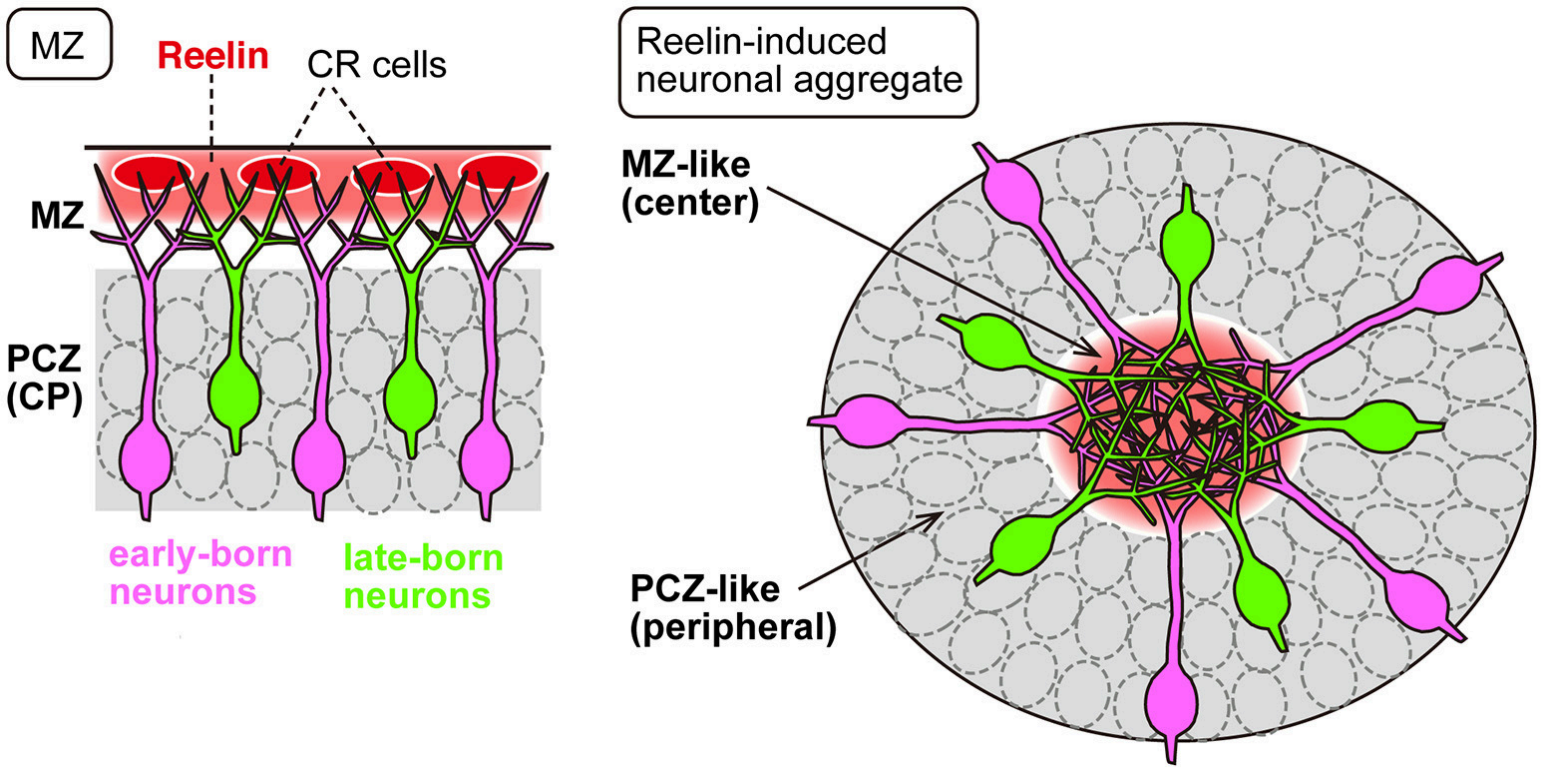
**Ectopic expression of Reelin causes neuronal aggregation *in vivo***. In the developing neocortex **(Left)**, Reelin (red) is mainly secreted from the CR cells in the MZ, into which the migrating neurons extend their apical leading processes. The PCZ is densely occupied by neuronal somata (gray). In neuronal aggregation induced by ectopically expressed Reelin **(Right)**, the leading processes of the migrating neurons assemble in the Reelin-rich central region, and the cell bodies are aligned in the PCZ-like peripheral region (gray). In both cases, the migrating neurons exhibit an “inside-out” cell arrangement in which the late-born cells (green) pass by the early-born neurons (magenta).

Although the results of ectopic Reelin expression described above (Kubo et al., 2010) were intriguing in terms of the cell alignment profile, it was not clear whether Reelin directly promoted adhesion among neurons or rather caused them to form aggregates as a result of being repelled by the surrounding cellular environment. A more recent study showed that Reelin directly promoted adhesion among dissociated neurons in culture (Matsunaga et al., 2017). Moreover, direct measurement of the adhesive forces revealed that Reelin promotes neuronal adhesion to N-cadherin during the aggregate formation (Matsunaga et al., 2017). Consistent with this, inhibition of N-cadherin impaired the neuronal aggregate formation induced by Reelin both *in vivo* and *in vitro*. Interestingly, the Reelin-induced enhancement of N-cadherin-dependent adhesion occurs only transiently (Matsunaga et al., 2017). These results clearly indicate that Reelin directly promotes adhesion among neurons during the aggregate formation. Future studies on the molecular and cellular mechanisms underlying the dynamic changes of N-cadherin-mediated cellular adhesiveness induced by Reelin may be expected to provide a more precise understanding of how appropriate layering of the neocortical neurons is regulated.

What is the physiological significance of the transient neuronal accumulation process observed in the PCZ? One possibility is that the neuronal aggregate itself might produce the physical force needed for the neurons to enter the PCZ. Since the PCZ is already densely packed with the neurons that arrived earlier, the subsequently arriving neurons might need to undergo some cellular changes, such as in their adhesive properties or in the cytoskeletal structure, to intercalate into the crowded environment. Consistent with this idea, migrating neurons start to express various genes when they arrive beneath the PCZ (Tachikawa et al., 2008). Or, neuronal aggregation might help in properly terminating radial migration at the border between the CP and MZ. Previous reports have revealed that the termination of neuronal migration is strictly regulated by the Reelin signal. When neurons reach the top of the CP, Reelin induces degradation of Dab1 via a SOCS7-Cul5-Rbx2-mediated proteasome pathway to inhibit further migration (Arnaud et al., 2003; Feng et al., 2007; Simo et al., 2010; Simo and Cooper, 2013). Multiple lines of evidence have demonstrated that Reelin signaling is required for polarized neurite growth (Niu et al., 2004; Olson et al., 2006; Jossin and Goffinet, 2007; Matsuki et al., 2010; Miyata et al., 2010; Nichols and Olson, 2010; Jossin and Cooper, 2011; O'Dell et al., 2012). A recent study has suggested that Reelin signaling controls migration arrest via polarized dendritogenesis. O'Dell et al. showed, using multiphoton time-lapse imaging, that in *reeler* cortices, a subset of neurons retracted and reorganized their arbors in an abnormal tangential direction away from the MZ upon completing migration (O'Dell et al., 2015). They also showed that the application of exogenous Reelin to *reeler* explants restored the polarized dendritogenesis, suggesting that Reelin-dependent dendritogenesis is closely related to the migration arrest beneath the MZ (O'Dell et al., 2015). Consistent with this, Reelin receptors are localized in the apical dendrites in the MZ (Hirota et al., 2015). A previous report showed that a large number of cortical neurons exist in the MZ of adult *Vldlr* KO mice, but not in that of *Apoer2* KO mice, suggesting that Vldlr is important for termination of the radial migration of neurons beneath the MZ (Hack et al., 2007). Another recent study reported observation of the overmigration phenotype in neonatal *Apoer2* KO mice (Hirota et al., 2016). Ectopic neurons in the MZ of the *Apoer2* KO mice might be eliminated from the MZ by cell death in the later postnatal stages. Together, accumulating evidence indicates that the Reelin signal is important for termination of the radial migration of neurons in the developing brain. It is important to determine, in a future study, how transient neuronal accumulation in the PCZ contributes to the completion and termination of neuronal migration.

## Future directions

In summary, Reelin signaling controls multiple steps of neuronal migration through shared and distinct molecular mechanisms. Reelin signaling has been shown to be involved in LIMK1/n-cofilin activation during somal/terminal translocation and migration in the IZ. Reelin-dependent regulation of the adhesion molecule N-cadherin plays important roles in somal/terminal translocation, multipolar-bipolar transition and cell aggregation. Reelin signaling is also involved in the activation of integrin α5β1, another adhesion molecule, during terminal translocation.

While various downstream key components of Reelin signaling have been identified, it still remains unknown how Reelin signaling controls the multiple processes of neuronal migration and layer formation in the developing brain. Defect in neuronal migration is known to be associated with several neurological disorders, including epilepsy and mental retardation. Lissencephaly is one of the severe developmental disorders that may result from impaired neuronal migration, and is characterized by complete or incomplete agyria of the cerebral cortex. Lissencephaly and cerebellar hypoplasia, a similar phenotype to that observed in the *reeler* mutant mice, have been observed in human patients carrying *reelin* mutations (Hong et al., 2000), suggesting that in both rodents and humans, dysregulated Reelin signaling causes neurological defects. Thus, molecules involved in Reelin signaling could be potential targets for therapeutic intervention against neurological disorders. Further studies of the precise mechanisms underlying the functions of Reelin in various processes of migration of neurons in the developing brain, including neuronal adhesion and aggregation, would increase our understanding of mammalian neocortical development under both physiological and pathological conditions.

## Author contributions

All authors listed, have made substantial, direct and intellectual contribution to the work, and approved it for publication.

### Conflict of interest statement

The authors declare that the research was conducted in the absence of any commercial or financial relationships that could be construed as a potential conflict of interest.
